# Dynamic Load Balancing Data Centric Storage for Wireless Sensor Networks

**DOI:** 10.3390/s101110328

**Published:** 2010-11-17

**Authors:** Seokil Song, Kyoungsoo Bok, Yun Sik Kwak, Bongeun Goo, Youngsik Kwak, Daesik Ko

**Affiliations:** 1 Department of Computer Engineering, Chungju National University, 72 Daehak-ro, Chungju-si, Chungbuk 380-702, Korea; E-Mails: yskwak@cjnu.ac.kr (Y.S.K.); bggoo@cjnu.ac.kr (B.G.); 2 (C) Gain IT/ #503, 505, Daejeon Intelligent Robot Engineering Center, 629 Tamnip-dong, Yuseong-gu, Daejeon, Korea; E-Mail: ksbok@gainit.co.kr (K.B.); 3 Department of Venture and Business, Jinju National University, 150 Chilam-dong, Jinju-si, Kyeongnam 660-758, Korea; E-Mail: pricing2@paran.com (Y.K.); 4 Department of Electronics Engineering, Mokwon University, Mokwon Gil 21, Seo-gu, Daejeon, 302-318, Korea; E-Mail: kds@mokwon.ac.kr (D.K.)

**Keywords:** sensor network, data centric storage, index

## Abstract

In this paper, a new data centric storage that is dynamically adapted to the work load changes is proposed. The proposed data centric storage distributes the load of hot spot areas to neighboring sensor nodes by using a multilevel grid technique. The proposed method is also able to use existing routing protocols such as GPSR (Greedy Perimeter Stateless Routing) with small changes. Through simulation, the proposed method enhances the lifetime of sensor networks over one of the state-of-the-art data centric storages. We implement the proposed method based on an operating system for sensor networks, and evaluate the performance through running based on a simulation tool.

## 1. Introduction

Recently, wireless sensor networks have attracted great interest since they provide us with a means for continuous and real-time monitoring and interacting with the physical world. The sensor nodes that make up the sensor network continuously collect physical data such as location, CO_2_, temperature, humidity and so on. The collected data is stored in sensor networks, or they are transmitted to a base station for further query processing. Many studies have proposed techniques for energy efficient data storage and query processing in sensor networks. Among them, data centric storage (DCS) is one of the most interesting techniques [1].

Data centric storage (DCS) stores data in a sensor network by its values. Each sensor reading (event) is mapped to an owner sensor node by a hashing function based on the values of the event’s attributes. The event is routed to the owner node from the original sensor node according to some routing protocols, such as greedy perimeter stateless routing (GPSR) [2]. Therefore, all events with the same value are stored at the same owner node.

Existing DCSs suffer the lack of a strategy for handling hot-spots. A storage hot-spot occurs when many events are mapped to a small number of sensor nodes. Due to the storage constraints of sensor nodes, a storage hot-spot increases the dropping rate of events by overloaded sensor nodes. Queries for events in a storage hot-spot may be delayed due to contention at the storage sensor nodes and the surrounding sensor nodes. The sensor nodes in the hot-spot may quickly consume energy, due to the high load of query and storing sensor readings. Increased dead sensor nodes results in decreasing coverage area, and causes the formation of coverage gaps within such areas.

In distributed index for multi-dimensional data (DIM) [3] which is one of the better-known DCSs, events are mapped to sensor nodes based on a K-D tree. The union of its leaf nodes covers the whole sensor network area and a leaf node contains one or zero sensor nodes. The bisection history of a leaf node is transformed to the address of a sensor node contained in the leaf node. When a sensor node generates an event, it maps the event to an address based on a repetitive fixed uniform splitting of its attributes’ ranges in a round robin fashion. The event is transmitted to the owner node using GPSR. DIM has two major problems. One problem is that there may exist orphan regions that contain no sensor nodes. The nodes contained in the neighbour regions of orphan regions have to take care of the load for the orphan regions. The other is the storage hotspot problem discussed above.

K-D tree based data centric storage (KDDCS) [4] was proposed to solve the problems of DIM. In KDDCS, to avoid orphan regions, a region is divided up so that both partitioned regions contain the same numbers of sensors nodes if possible. Consequently, a KDDCS K-D tree is balanced and there are no orphan regions. However, according to this feature, KDDCS modifies GPSR to use it as its routing technique. The modified GPSR may require more communication costs to send one message than the original GPSR.

In addition, to solve the storage hot-spot problems, KDDCS presents a K-D tree re-balancing (KDTR) algorithm. However, the KDTR requires extra communication costs to move data from a region to its neighbour region for load balancing. Besides DIM and KDDCS, various other DCSs have been proposed.

In [5], zone partitioning (ZP) and zone partial replication (ZPR) are proposed. ZP partitions the hot spot storage responsibility among a larger number of sensors, while ZPR replicates the hot spot in neighbours. The authors of [6] present dynamic balanced data-centric storage (DBAS) that uses a cooperative strategy between the base station and the in-network processing. Virtual grid based DCS is proposed in [7]. It uses a virtual grid to assign addresses to sensor nodes and distribute the load of a hot-spot area by using a multilevel grid technique. In fact, this paper is an extended version of [7].

In this paper, we propose a new data centric storage based on multi-level grid techniques. The proposed DCS divides a sensor network area with virtual grid techniques, so all cells cover the same size of area. The proposed DCS uses GPSR to send an event or query to a target with some modifications, but the modified GPSR does not require any extra communication costs. Also, it distributes the load of a hot spot sensor node by using multilevel grid technique without moving events.

This paper is organized as follows. Section 2 gives the description of DIM and KDDCS. Section 3 presents the proposed multi-level grid based DCS. Experimental results are discussed in Section 4, and we conclude in Section 5.

## 2. Related Work

The advantages of DCS have become well known through some studies [1,8]. In [3] a DIM index, which is a distributed index for multi-dimensional data was proposed. Usually, a sensor network has multi-dimensional data such as humidity, temperature, illumination and so on. A DIM indexes the multi-dimensional data in a K-D tree structure by the data values to speed up the multi-dimension range queries, and routes the data to the exact sub-tree using the GPSR routing protocol.

**Figure 1.**
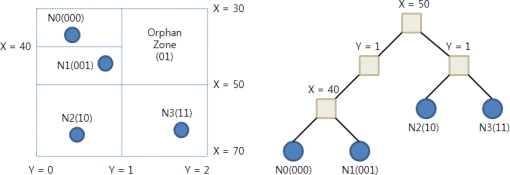
An example of DIM.

Each sensor node in DIM determines its node address by uniformly splitting the sensor network area in a round robin fashion. A sensor node splits the area horizontally, then vertically, and shifts its bit code to the left with every split by 0 b (or 1 b) when it falls above (or below) the horizontal split line. It also shifts its bit code to the left by a 0 bit if falling on the left of the vertical split line, or 1 bit otherwise. This process ends when every sensor lies by itself in a zone. Then the bit code becomes the address of the sensor node. Thus, the length of the binary address of each sensor (in bits) represents its depth in the underlying K-D tree. Figure 1 shows the K-D tree that the DIM forms for a simple network.

In case any orphan zones exist (zones physically containing no sensors in their geographic area), the ownership of each of these zones is delegated to one of its neighbour sensors. In Figure 1, the orphan zone 01b is delegated to the sensor node N. When a sensor node detects an event, it generates an event bit-code as follows. The bit-code generation proceeds in rounds. There is a range R_j_ associated with each attribute j of the event. Initially, the range R_j_ is the full range of possible values for attribute j. Round i, determines the (i + 1)^th^ high order bit in the code. Round i depends on attribute j = i mod k of the event, where k is the number of attributes in the event. Assume that the current value of R_j_ is [a, c], and let b = (a + c)/2 be the midpoint of the range R_j_. If the value of attribute j is in the lower half of the range R_j_, that is in [a, b], then the i^th^ bit is 0 b, and R_j_ is set to be the lower half of R_j_. If the value of attribute j is in the upper half of the range R_j_, that is in [b, c], then the i^th^ bit is 1, and R_j_ is set to be the upper half of R_j_.

For example, in Figure 1, events are composed of two attributes which are X and Y, with ranges (30, 70) and (0, 2), respectively. If N3 detects an event (55, 0.6), it generates a bit-code for the event as follows. X is in the top half of the range [30, 70] so the first bit is 1, Y is in the bottom half of the range [0, 2] so the second bit is 0b, then X is in the bottom half of the range [50, 70] so the third bit is 0 b, and finally, Y is in the top half of the range [0, 1] so the fourth bit is 1b. Then, the event will be routed toward the geometric location specified by code 1001b through GPSR.

A major problem of DIM is the lack of handing of hot-spots. The presence of a hot-spot leads to an increased energy consumption rate due to overloaded sensors. More critically, the sensors in and near the hot-spot may quickly run out of energy, due to the high query load imposed on them (in addition to event insertions). These results in a loss of the events generated at these sensors, the events stored at these sensors, and possibly a decrease in network connectivity. Increased death of sensors results in decreasing coverage area and causes the formation of coverage gaps within such an area.

Several techniques [4,5,9] have been proposed to address load balance problems on DIM. In [9], a Zone Sharing scheme is proposed to locally detect hot-spots and try to evenly distribute their loads among the sensor nodes in the network. In [5], Zone Partitioning (ZP) and Zone Partial Replication (ZPR) are proposed. ZP is based on partitioning the hot-spot storage responsibility among larger number of sensors, while ZPR is based on replicating the hot-spot in sensor neighbouring the hot-spot area.

KDDCS proposed in [4] is also based on a K-D tree-like DIM so the construction process of K-D tree and the process of mapping an event to a bit-code are similar to those in DIM. However, in KDDCS, the refinement of regions in the formation of the K-D tree has the property that the numbers of sensors on both sides of the partition are approximately equal. As a result of this, K-D tree of KDDCS will be balanced, while the K-D tree of DIM is unbalanced so there will be no orphan regions. Also, regardless of the geographic distribution of the sensors, the ownership of events will uniformly distributed over the sensors if the events are uniformly distributed over the range of possible events.

As described earlier, the bit-code for an event in DIM means a geographic location, so DIM can use GPSR routing to transmit the event to an owner node. However, the K-D tree in KDDCS splits the sensor network region so as to distribute sensor nodes equally. This means that the bit-code for sensor nodes and events cannot represent the geographical locations. Therefore, KDDCS presents a modification of GPSR routing, namely Logical Stateless Routing (LSR), for the routing of events from their generating sensors to their owner sensors, that is competitive with the GPSR routing used in DIM.

**Figure 2.**
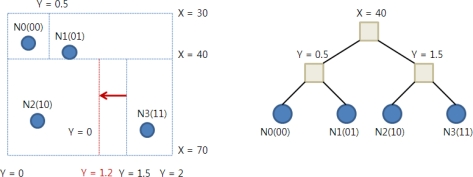
An example of KDDCS.

Figure 2 shows a K-D tree for the same sensor network of Figure 1 built by KDDCS. In this figure, there is no orphan zone and the K-D tree is well balanced. KDDCS also has a load balancing algorithm called K-D Tree Re-balancing (KDTR). The re-balancing algorithm guarantees load balance even if the event distribution is not uniform. If a sensor node detects a hot-spot, it changes the split lines to re-balance the sub-tree. For example, in Figure 2, if the load of N2 exceeds a certain threshold value, it changes the split line from Y = 1.5 to Y = 1.2 and moves some events from N2 to N3.

Dynamic balanced data-centric storage (DBAS) [6] presents a cooperative strategy between the base station and the in-network processing. It takes advantage of the resources of the base station to optimize the storage. Furthermore, DBAS is not based on the DIM scheme, so it does not have to maintain complex index structures like K-D trees. The re-balancing operation of DBAS is easy because no data needs to be transferred to other sensor nodes. However, in DBAS, sensor nodes must cooperate with the base station to maintain data centric storage.

## 3. Proposed Data Centric Storage

### 3.1. Initial Process

The proposed DCS is based on virtual multilevel grid techniques. The geographic area for a sensor network is partitioned into *2^b^* rectangular cells, where b denotes the user specified number of bits. In our method, the sensor network area is recursively divided into four quadrant cells according to the given level. Once we have divided the sensor network space in this way, we assign a unique bit-string of length b to each cell on each level. The bit-string for each cell on a level is determined by the Z-order. The bit-string of a lowest level cell is assigned to sensor nodes that are included the cell. The bit-string of an upper level cell is calculated by shifting the bit-string of any contained lower cell to the right two times.

**Figure 3.**
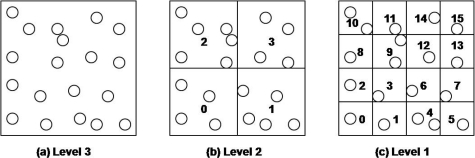
Grid based index when the given level is 3.

In a cell, there may be more than two sensor nodes. In this case, one of them becomes the cell header (CH). The cell header is selected by sensor nodes in the cell and manages other cell sensor nodes. Sensor nodes construct this virtual multilevel grid by themselves. When the network is deployed, the grid information such as the geographic area of sensor network, and the level and the number of bits b is broadcasted to the sensor network, or is given by sensor network manager during the deployment of sensor nodes. Also, it is assumed that the nodes are aware of locations of themselves and their neighbor nodes within their radio ranges. Each sensor node calculates, and assigns by itself a cell id (CID) according to its location. Figure 3 shows an example when the given level is 3. Cells on each level have their own unique numbers.

### 3.2. Data Insertion of GDCS

When a sensor node detects an event (sensor reading), it forwards the event to the center point of a cell that is selected by mapping the event's value to a lowest level CID. A data packet consists of the event, the coordinate of the cell's center point and the CID of the cell. We use GPSR algorithm with a simple modification to route the data packet to the target sensor node. We add an end condition to the original GPSR when the forwarding action is stopped. Whenever a sensor node receives a packet, first, it compares the CID of the packet with its CIDs. If the CIDs are the same, the sensor node stores the data of the packet. Otherwise, it performs the forwarding actions according to the GPSR.

In our method, storage hot-spot problem can still occur. If relatively many events are mapped to a certain cell id, the sensor nodes in the cell will be overloaded. Consequently, some events are lost, and the sensor nodes run out of energy more quickly. Our proposed method uses multilevel grid techniques to handle the storage hot-spot problem. If a hot-spot cell on the lowest level is detected, the cell and its neighbor cells on the same level are merged into a higher level grid cell. A new cell id on the higher level is assigned to the sensor nodes that are contained in the higher cell. After that merging process, the work load of the hot spot cell is distributed to all of the sensor nodes of the merged cell.

The merging process is performed by the sensor nodes themselves. When a CH detects that insertion work load of its cell is increased over the predefined threshold, the merging process is started. The CH calculates the cell id at the higher level, and sends the cell id to sensor nodes that are contained in a cell at the higher level.

**Figure 4.**
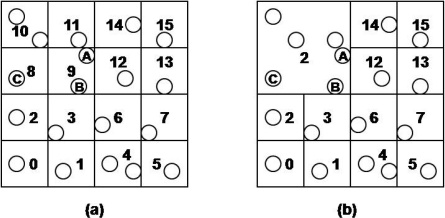
Merge operation for handling hot-spots.

For example, in Figure 4(a) the CH of sensor nodes of cell 9 detects that the cell is hot spot. Then, the CH merges 8, 9, 10 and 11 cells to the higher level cell, 2 on level 2 to distribute its insertion and query processing load to neighbor sensor nodes. In the following, we denote *CID* (*L*) as *CID* on level *L*. In order to merge the cells, the CH sends merge messages to sensor nodes contained in the cell 2, and the sensor nodes that received the message assign new cell id 2 to them. Subsequently, sensor nodes in the hot spot cell have the newly assigned *CID*, and sensor nodes in the merged cell have two *CID*s on the different levels. Since the new cell is created, a new CH must be selected. At the first time, the CH of hot spot cell becomes the new CH of 2. Then, the CH is reselected according to the energy consumption of sensor nodes later.

Events are inserted into sensor networks as follows. When a sensor node detects an event (sensor reading), it maps the event to a cell id at the lowest level. Then, it forwards a packet that consists of the event, the cell id and the geographic location to the center point of the cell. A sensor node that receives the packet compares the cell id of the packet and its cell ids. If they are matched, the packet is forwarded to the CH of the sensor node. The CH knows the energy consumption, storage utilization, values of stored events of sensor nodes, so it can select a proper sensor node to store the data of the packet, and sends the packet to the sensor node.

If a sensor node has two or more cell ids of different levels, first, the cell id on the lowest level is compared with the received packet's cell id, and then the cell ids on the higher level are compared repeatedly until finding a matched cell id. If the level of the sensor node’s cell id is higher than that of the received packet, the sensor node converts the received cell id to the level of its cell id before comparing them by shifting bits. Algorithm 1 shows the event insertion algorithm.

A CH also consumes energy more quickly since it manages the sensor nodes and every insertion in a cell. Therefore, a CH of a cell should be reelected by considering the energy level of each sensor node in the cell. Since the CH knows the energy level of each sensor node, the CH can select the most proper sensor node as a new CH.

**Figure 5.**
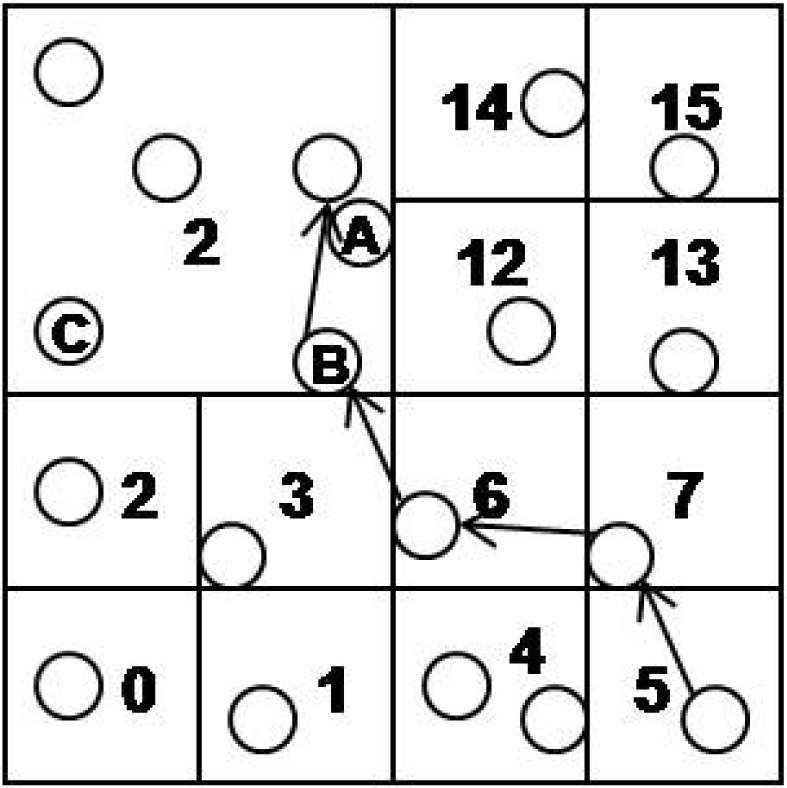
Example of insertion.

For example, in Figure 5, we assume that the sensor node in cell 5 has an event *e* and the lowest level cell id of *e* is 10. Also, sensor node A has cell ids 9(1) and 2(2). The numbers, 3 and 2, in parenthesis mean the level of cell ids. Sensor node B and C have cell ids 9(1), 2(2) and 8(1), 2(2), respectively. All sensor nodes in cell 2(2) know that cell 9(1) requested a merge operation and the node B is the cell header. The sensor node in cell 5 starts to route the event *e* to cell 10. At that time, the sensor node does not know that the cell 10(1) is merged to 2(2). *e* is routed to the node B through the nodes in the cell 7(1) and 6(1). B has two cell ids, 9(1) and 2(2), so first we compare 9(1) with e’s cell id 10(1). They are not matched, so we get the cell id of level 2 from the *e*’s cell id 10(1). It is simply calculated by shifting 2 bits of 10(1010 b) to right. The cell id of level 2 is 2(10 b) and we compare 2(2) with *e*’s 2(2). They are matched, so we can insert *e* to the cell 2(2). CH of the cell 2(2) determines *A* as the most proper node, and we insert the *e* into the node *A*.

**Algorithm 1.** Insertion algorithm.

/*
Function handle_event () is a call back function.
It is called whenever an event is occurred.
*/
handle_event (event)
{
if event is a new sensor reading
make a packet (event, target_cid, target_xy);
send packet to a neighbor cell according to GPSR algorithm;
end handle_event;
end if
if event is a received packet
for (each current_node.cell_ids)
if (packet.cell_id is matched with one of current_node.cell_ids)
if (current_node is CH)
send packet to a proper node in current_node's cell ;
else
send packet to CH;
end if
end handle_event;
end if
end for
send packet to a neighbor cell according to GPSR algorithm;
else
end if
}

## 4. Performance Evaluation

We performed a simulation to evaluate the performance of the proposed DCS. We assume that 400 nodes are randomly deployed in a square area, 200 × 200 m^2^. Also, we assume that each sensor node has initial energy of 1,000 units and a storage capacity of 100 units. A message transmission from a sensor node to its neighbor node consumes 0.1 energy units for sending the message and 0.05 energy units for receiving the message. The radio range each node has is 15 m. Every node generates 50 events that are normal distribution of values, and a percentage of 80% of the events falls into a percentage of 20% of the reading range. We measure the energy level of each sensor node, and compare the results with that of KDDCS which is one of the most well-known data centric storage. Table 1 shows the simulation parameters.

Table 2 shows the average energy level of each sensor node in the hot-spot area and entire area. As shown in the table, entire sensor nodes of the KDDCS consume more energy than those of the proposed DCS by about 73%. In the hot-spot area, sensor nodes of KDDCS consume much more energy than those of the proposed DCS by about 97%. The reasons why the proposed GDCS outperforms KDDCS are as follows. The first reason is that the KDDCS needs to visit the node that contains the split information to forward the packet to the next node. Also, in a KDDCS’ hot spot handling strategy, a node in the hot spot moves data to neighbor nodes whenever adapt the area covered by a sensor node.

In this paper, we implement a simple application that uses our GDCS based on TinyOS [10]. Then, we run the implemented application based on TOSSIM [11] to show how our GDCS distributes the workload of a hot spot area. Table 3 shows the TOSSIM parameters. The number of nodes is 256 and the width and height of the sensor network are was 100 m. The radio range is 10 m and the number of bits per grid is 8.

When we set the number of levels as 1, *i.e.*, multilevel grid techniques are not used, as shown in Table 4, the sensor nodes of the hot spot area receive 40% of the total messages. However, when the number of levels was 2, the ratio of the messages of sensor nodes in hot spot area to the total message is decreased to about 21%. The results mean that the proposed GDCS distributes the workload of hot spot areas effectively.

## 5. Conclusions

In this paper, we have proposed a grid technique based data centric storage for sensor networks. The proposed DCS (GDCS) handles storage hot spot problems by using multilevel grid techniques. Each cell header detects a hot spot, and lowers the grid level of the hot spot area so more sensor nodes cooperate to store data and to process queries. We have performed simulations to evaluate the GDCS. The GDCS improved the life time of sensor networks by about 60% over KDDCS. We also showed that the proposed GDCS efficiently distributed the workload of hot spots.

## Figures and Tables

**Table 1.** Parameters for performance evaluation.

Number of Sensor Nodes	400
Sensor Network Area	200 × 200 m^2^
Initial Energy of a Sensor Node	1,000 units
Energy Consumption per Transmit	0.1 units
Energy Consumption per Receive	0.05 units
Storage Capacity	100 units
Radio Range	15 m

**Table 2.** Average energy level of each sensor node.

Energy Level	KDDCS	GDCS

Average Energy Level of a Sensor Node	752.38 units	857.69 units
Average Energy Level of a Sensor Node in Hot-spot	331.13 units	611.01 units

**Table 3.** Parameters for running TOSSIM.

Number of Nodes	256
Sensor Network Area	100 × 100 m^2^
Radio Range	10 m
b	8 (Number of Cells is 256)

**Table 4.** Message receiving rate.

	
	GDCS (Level 1)	GDCS (Level 2)

Message Receiving Rate	40%	21%
